# Supplementary material for: Comparing Functional Connectivity Matrices: A Geometry-Aware Approach applied to Participant Identification
Source: Neuroimage. Author manuscript; Available in PMC 2021 Feb 15. (PMC6995445; doi:10.1016/j.neuroimage.2019.116398)
Supplement: 1 [file NIHMS1062336-supplement-1.pdf]

bioRxiv preprint first posted online Jul. 2, 2019; doi: http://dx.doi.org/10.1101/687830. The copyright holder for this preprint (which was not peer-reviewed) is the author/funder, who has granted bioRxiv a license to display the preprint in perpetuity. It is made available under a CC-BY-NC-ND 4.0 International license.

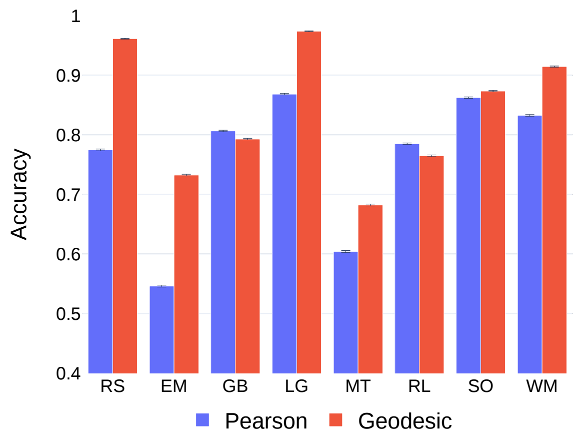

Fig. S1: Participant identification for the eight conditions using the geodesic distance and Pearson dissimilarity. Training and testing data were from the same condition. Fixation period or "mini resting periods" were not trimmed from runs of task data (as done in Fig. 3). Abbreviations: EM, emotion processing; GB, gambling; LG, language; MT, motor; RL, relational processing; RS, resting-state; SO, social cognition; WM, working memory.

Supplemental material

S1. Identification accuracy when runs were not trimmed

In the main body of the text, to ensure that only task-related segments of a run were retained, "mini resting periods" in the form of fixation periods were removed (see Section 2.1). We repeated our analysis without trimming runs of task data using whole-cortex FCs. Identification accuracy for each condition is shown in Fig. S1. Accuracy obtained using the geodesic distance exceeded that of Pearson dissimilarity for all conditions except the *gambling* and *relational* tasks ($p = 1$ for *gambling* and *relational* tasks, $p < 10^{-6}$ for all tasks; reference $\alpha = 0.05/8 = 0.00625$ given 8 conditions; Fig. S2). The mean improvement using geodesic distance was around 8% (as high as 18% on *resting-state* data).

S2. Effect of global signal regression on identification

We repeated our analysis by including global signal regression (GSR) in the preprocessing pipeline for resting-state data [20, 23]. The use of GSR is still debated [26] and can potentially spread underlying group differences to regions that may never have had any [31]. We limit our analysis in this section to *resting-state* data; we did not include GSR in the preprocessing pipeline for results in the main text. In the data employed (see Acknowledgements), 8 subjects' data were removed because they did not not pass quality control check [23]. Thus, the results reported this section were based on $N = 92$ participants. We performed participant identification using whole-cortex FCs. Regardless of the inclusion of GSR in preprocessing, identification accuracy improved using geodesic distance compared to Pearson dissimilarity (Fig. S3A). However, using GSR improved accuracy for both measures. When segments of smaller lengths were extracted from *resting-state* data, accuracy improved using geodesic distance for all segment lengths (Fig. S3B). When GSR was used, accuracy using geodesic distance was close to 95% with only 200 time points (compared to 70% without GSR; Fig. S3C).

S3. Effect of number of ROIs in the parcellation on identification

To study the effect of the parcellation scheme on participant identification accuracy using the two measures, we employed various parcellations with ROIs ranging from a 100 to 400. In general, mean participant identification accuracy increased with increase in ROIs indicating that finer resolution or detail in the FC revealed more uniqueness. Mean accuracy using the geodesic distance was consistently higher than the mean accuracy using Pearson dissimilarity. For several conditions (*resting-state*, *language*, *motor*), accuracy using geodesic distance on FCs obtained with 100 ROIs was greater than accuracy obtained using Pearson dissimilarity with 400 ROIs (Fig. S5).

S4. Computing geodesic distances for matrices without full rank

Computing the geodesic distance between two FC matrices Q_1 and Q_2 (Equation 3) requires Q_1 to be invertible, or equivalently, all the eigenvalues of Q_1 must be strictly greater than zero. When FC matrices are based on n ROIs and n is larger than number of frames in the run, the *rank* of the resulting FC matrix is not full (i.e., $< n$), and some of its some eigenvalues are equal to 0. In practice, when the number of ROIs $n <$ (0.9 × number of frames), we applied the procedure below to ensure full rankness.

To handle such cases, we adopted a simple approach here: we added the identity matrix I to both Q_1 and Q_2, causing the eigenvalues of the correlation matrices of interest to be increased by 1. Because all eigenvalues are then greater than 0, the matrices are invertible. In such cases, the geodesic distance, $d_G(Q_1+I, Q_2+I)$, serves as a proxy for the geodesic distance between the two matrices. Note that the scenario of low-rank FC matrices arises only for whole-cortex analysis, as for the subnetwork analyses, the number of ROIs in question was always greater than the number of frames in the run.

For reference, the procedure above was employed in the following cases: whole-cortex results for all tasks; whole-cortex *resting-state* results with lengths less than 400 TRs; and whole-cortex results involving trimmed data.

References

[1] Aggarwal, C.C., Hinneburg, A., Keim, D.A., 2001. On the surprising behavior of distance metrics in high dimensional space, in: International conference on database theory, Springer. pp. 420–434.

[2] Allen, E.A., Damaraju, E., Plis, S.M., Erhardt, E.B., Eichele, T., Calhoun, V.D., 2014. Tracking Whole-Brain Connectivity Dynamics in the Resting State. Cerebral Cortex 24, 663–676. doi:10.1093/cercor/bhs352.

bioRxiv preprint first posted online Jul. 2, 2019; doi: http://dx.doi.org/10.1101/687830. The copyright holder for this preprint (which was not peer-reviewed) is the author/funder, who has granted bioRxiv a license to display the preprint in perpetuity. It is made available under a CC-BY-NC-ND 4.0 International license.

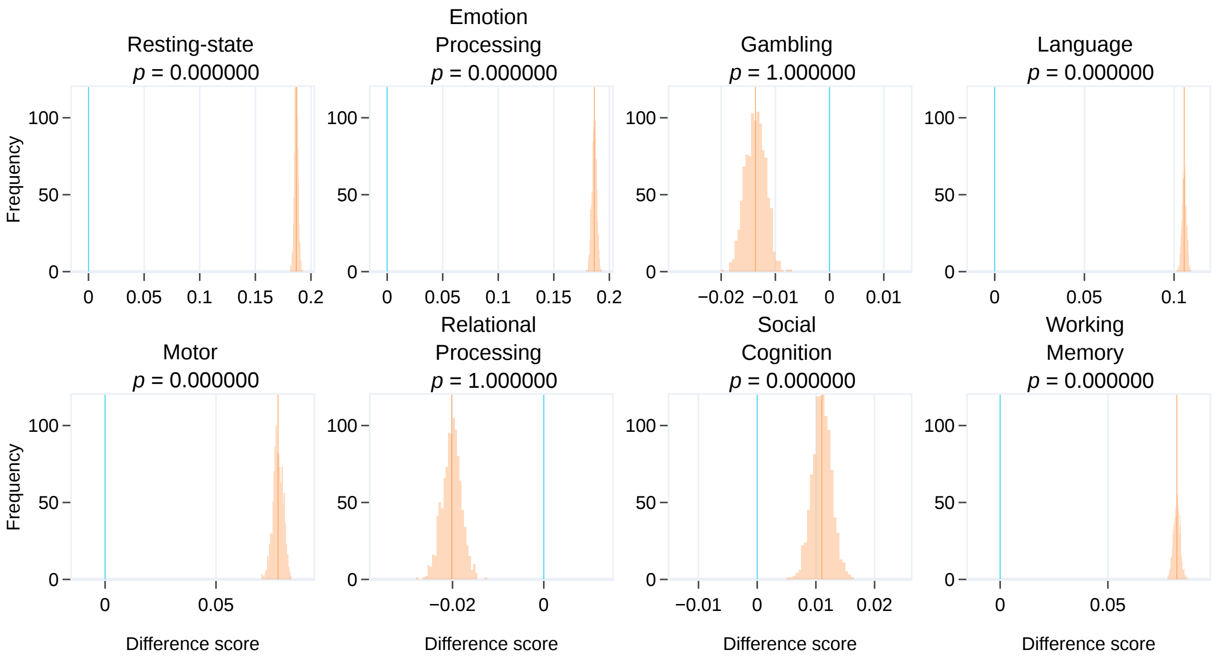

Fig. S2: *Whole-cortex FCs without trimming runs:* Comparison of identification accuracy based on geodesic distance and Pearson dissimilarity for each condition. Identification was based on whole-cortex FCs. Runs were not trimmed as in the main body of the work (see Section 2.1). For each condition, the distributions shown in orange represent the difference between the mean participant identification accuracy using the geodesic distance and Pearson dissimilarity across the outer bootstrap iterations (see Section 2.7). The orange line indicates the mean of the difference distribution and the blue line indicates zero difference.

[3] Amico, E., Goñi, J., 2018. The quest for identifiability in human functional connectomes. Scientific reports 8, 8254.

[4] Amrhein, V., Greenland, S., McShane, B., 2019. Scientists rise up against statistical significance.

[5] Barch, D.M., Burgess, G.C., Harms, M.P., Petersen, S.E., Schlaggar, B.L., Corbetta, M., Glasser, M.F., Curtiss, S., Dixit, S., Feldt, C., Nolan, D., Bryant, E., Hartley, T., Footer, O., Bjork, J.M., Poldrack, R., Smith, S., Johansen-Berg, H., Snyder, A.Z., Essen, D.C.V., Consortium, f.t.W.M.H., 2013. Function in the human connectome: Task-fMRI and individual differences in behavior. NeuroImage 80, 169–189. doi:10.1016/j.neuroimage.2013.05.033.

[6] Boubela, R.N., Kalcher, K., Huf, W., Kronnerwetter, C., Filzmoser, P., Moser, E., 2013. Beyond noise: using temporal ica to extract meaningful information from high-frequency fmri signal fluctuations during rest. Frontiers in human neuroscience 7, 168.

[7] Boyd, S., Vandenberghe, L., 2004. Convex optimization. Cambridge university press.

[8] Cohen, M.S., 1997. Parametric analysis of fmri data using linear systems methods. Neuroimage 6, 93–103.

[9] Cortes, C., Vapnik, V., 1995. Support-vector networks. Machine learning 20, 273–297.

[10] Cox, R.W., 1996. Afni: software for analysis and visualization of functional magnetic resonance neuroimages. Computers and Biomedical research 29, 162–173.

[11] Elliott, M.L., Knodt, A.R., Cooke, M., Kim, M.J., Melzer, T.R., Keenan, R., Ireland, D., Ramrakha, S., Poulton, R., Caspi, A., et al., 2019. General functional connectivity: shared features of resting-state and task fmri drive reliable and heritable individual differences in functional brain networks. NeuroImage 189, 516–532.

[12] Essen, D.C.V., Smith, S.M., Barch, D.M., Behrens, T.E., Yacoub, E., Ugurbil, K., Consortium, f.t.W.M.H., 2013. The WU-Minn Human Connectome Project: An overview. NeuroImage 80, 62–79. doi:10.1016/j.neuroimage.2013.05.041.

[13] Estivill-Castro, V., 2002. Why so many clustering algorithms: a position paper. ACM SIGKDD Explorations Newsletter 4, 65–75. doi:10.1145/568574.568575.

[14] Finn, E.S., Scheinost, D., Finn, D.M., Shen, X., Papademetris, X., Constable, R.T., 2017. Can brain state be manipulated to emphasize individual differences in functional connectivity? NeuroImage 160, 140–151. doi:10.1016/j.neuroimage.2017.03.064.

[15] Finn, E.S., Shen, X., Scheinost, D., Rosenberg, M.D., Huang, J., Chun, M.M., Papademetris, X., Constable, R.T., 2015. Functional connectome fingerprinting: identifying individuals using patterns of brain connectivity. Nature neuroscience 18, 1664.

[16] Förstner, W., Moonen, B., 2003. A metric for covariance matrices, in: Geodesy-The Challenge of the 3rd Millennium. Springer, pp. 299–309.

[17] Glasser, M.F., Sotiropoulos, S.N., Wilson, J.A., Coalson, T.S., Fischl, B., Andersson, J.L., Xu, J., Jbabdi, S., Webster, M., Polimeni, J.R., Essen, D.C.V., Jenkinson, M., Consortium, f.t.W.M.H., 2013. The minimal preprocessing pipelines for the Human Connectome Project. NeuroImage 80, 105–124. doi:10.1016/j.neuroimage.2013.04.127.

[18] Gonzalez-Castillo, J., Hoy, C.W., Handwerker, D.A., Robinson, M.E., Buchanan, L.C., Saad, Z.S., Bandettini, P.A., 2015. Tracking ongoing cognition in individuals using brief, whole-brain functional connectivity patterns. Proceedings of the National Academy of Sciences 112, 8762–8767. doi:10.1073/pnas.1501242112.

[19] Hansen, E.C., Battaglia, D., Spiegler, A., Deco, G., Jirsa, V.K., 2015. Functional connectivity dynamics: Modeling the switching behavior of the resting state. NeuroImage 105, 525–535. doi:10.1016/j.neuroimage.2014.11.001.

[20] Kong, R., Li, J., Orban, C., Sabuncu, M.R., Liu, H., Schaefer, A., Sun, N., Zuo, X.N., Holmes, A.J., Eickhoff, S.B., et al., 2018. Spatial topography of individual-specific cortical networks predicts human cognition, personality, and emotion. Cerebral Cortex 29, 2533–2551.

[21] Kruskal, J.B., 1964. Multidimensional scaling by optimizing goodness of fit to a nonmetric hypothesis. Psychometrika 29, 1–27.

bioRxiv preprint first posted online Jul. 2, 2019; doi: http://dx.doi.org/10.1101/687830. The copyright holder for this preprint (which was not peer-reviewed) is the author/funder, who has granted bioRxiv a license to display the preprint in perpetuity. It is made available under a CC-BY-NC-ND 4.0 International license.

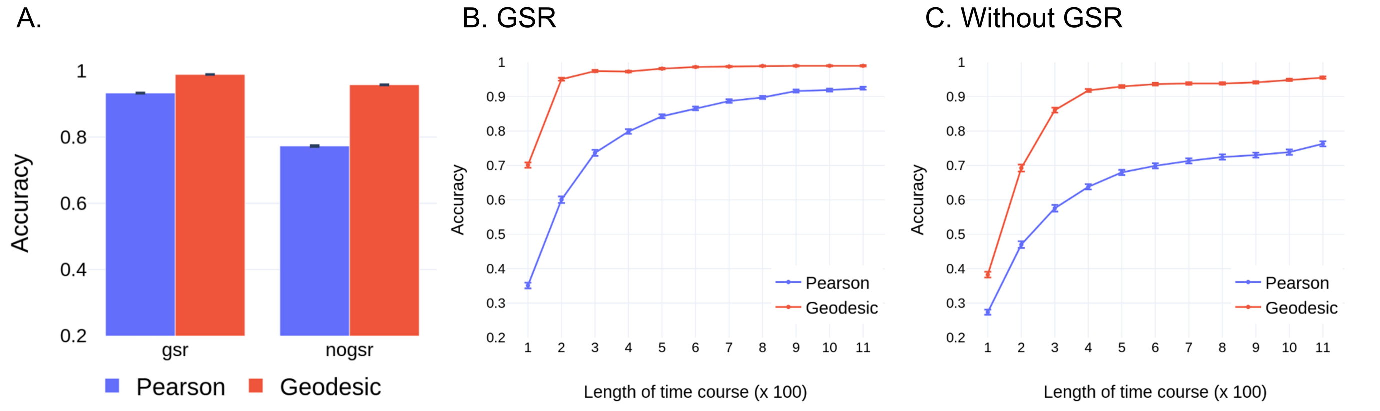

Fig. S3: A. Participant identification accuracy with global mean regression (GSR) included in the preprocessing pipeline (gsr) or not (nogsr). Accuracy using geodesic distance exceeded Pearson dissimilarity for both preprocessing methods. Participant identification accuracy as a function of segment length for *resting-state* data with GSR (in B) and without GSR (in C). In both cases, accuracy using geodesic distance exceeded Pearson dissimilarity at each segment length. Error bars indicate standard error of the mean across bootstrap iterations.

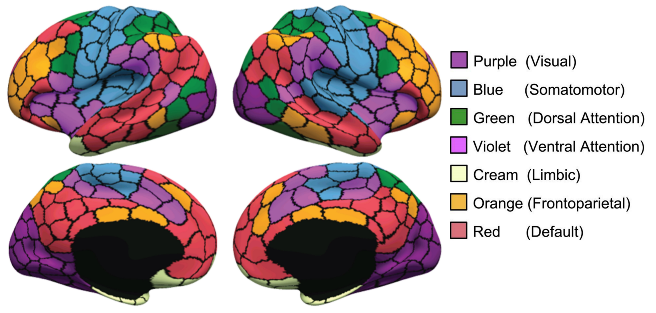

Fig. S4: Parcellation of the cortex into 300 ROIs as provided by [32]. ROIs were grouped into the 7 networks described in [39].

[22] Leonardi, N., Ville, D.V.D., 2015. On spurious and real fluctuations of dynamic functional connectivity during rest. NeuroImage 104, 430–436. doi:10.1016/j.neuroimage.2014.09.007.

[23] Li, J., Kong, R., Liegeois, R., Orban, C., Tan, Y., Sun, N., Holmes, A.J., Sabuncu, M.R., Ge, T., Yeo, B.T., 2019. Global signal regression strengthens association between resting-state functional connectivity and behavior. NeuroImage 196, 126–141.

[24] MATLAB, 2018. version 9.4.0.813654 (R2018a). The MathWorks Inc., Natick, Massachusetts.

[25] McShane, B.B., Gal, D., Gelman, A., Robert, C., Tackett, J.L., 2019. Abandon Statistical Significance. The American Statistician 73, 235–245. doi:10.1080/00031305.2018.1527253.

[26] Murphy, K., Fox, M.D., 2017. Towards a consensus regarding global signal regression for resting state functional connectivity mri. Neuroimage 154, 169–173.

[27] Ng, A.Y., Jordan, M.I., Weiss, Y., 2002. On spectral clustering: Analysis and an algorithm, in: Advances in neural information processing systems, pp. 849–856.

[28] Pennec, X., Fillard, P., Ayache, N., 2006. A riemannian framework for tensor computing. International Journal of computer vision 66, 41–66.

[29] Pessoa, L., 2014. Understanding brain networks and brain organization. Physics of life reviews 11, 400–435.

[30] Ponsoda, V., Martínez, K., Pineda-Pardo, J.A., Abad, F.J., Olea, J., Román, F.J., Barbey, A.K., Colom, R., 2017. Structural brain connectivity and cognitive ability differences: A multivariate distance matrix regression analysis. Human brain mapping 38, 803–816.

[31] Saad, Z.S., Gotts, S.J., Murphy, K., Chen, G., Jo, H.J., Martin, A., Cox, R.W., 2012. Trouble at rest: how correlation patterns and group differences become distorted after global signal regression. Brain connectivity 2, 25–32.

[32] Schaefer, A., Kong, R., Gordon, E.M., Laumann, T.O., Zuo, X.N., Holmes, A.J., Eickhoff, S.B., Yeo, B.T., 2017. Local-global parcellation of the human cerebral cortex from intrinsic functional connectivity mri. Cerebral Cortex 28, 3095–3114.

[33] Schultz, D.H., Cole, M.W., 2016. Higher intelligence is associated with less task-related brain network reconfiguration. Journal of Neuroscience 36, 8551–8561.

[34] Smith, S.M., Beckmann, C.F., Andersson, J., Auerbach, E.J., Bijsterbosch, J., Douaud, G., Duff, E., Feinberg, D.A., Griffanti, L., Harms, M.P., Kelly, M., Laumann, T., Miller, K.L., Moeller, S., Petersen, S., Power, J., Salimi-Khorshidi, G., Snyder, A.Z., Vu, A.T., Woolrich, M.W., Xu, J., Yacoub, E., Uurbil, K., Essen, D.C.V., Glasser, M.F., Consortium, W.M.H., 2013. Resting-state fMRI in the Human Connectome Project. NeuroImage 80, 144–168. doi:10.1016/j.neuroimage.2013.05.039.

[35] Sporns, O., 2010. Networks of the Brain. MIT press.

[36] Van Dongen, S., Enright, A.J., 2012. Metric distances derived from cosine similarity and pearson and spearman correlations. arXiv preprint arXiv:1208.3145 .

[37] Venkatesh, M., 2019. Online figures. https://github.com/makto-toruk/FC_geodesic.

[38] Xie, H., Calhoun, V.D., Gonzalez-Castillo, J., Damaraju, E., Miller, R., Bandettini, P.A., Mitra, S., 2018. Whole-brain connectivity dynamics reflect both task-specific and individual-specific modulation: A multitask study. NeuroImage 180. doi:10.1016/j.neuroimage.2017.05.050.

[39] Yeo, B.T.T., Krienen, F.M., Sepulcre, J., Sabuncu, M.R., Lashkari, D., Hollinshead, M., Roffman, J.L., Smoller, J.W., Zllei, L., Polimeni, J.R., Fischl, B., Liu, H., Buckner, R.L., 2011. The organization of the human cerebral cortex estimated by intrinsic functional connectivity. Journal of Neurophysiology 106, 1125–1165. doi:10.1152/jn.00338.2011.

[40] Zalesky, A., Breakspear, M., 2015. Towards a statistical test for functional connectivity dynamics. NeuroImage 114, 466–470. doi:10.1016/j.neuroimage.2015.03.047.

bioRxiv preprint first posted online Jul. 2, 2019; doi: http://dx.doi.org/10.1101/687830. The copyright holder for this preprint
(which was not peer-reviewed) is the author/funder, who has granted bioRxiv a license to display the preprint in perpetuity.
It is made available under a CC-BY-NC-ND 4.0 International license.

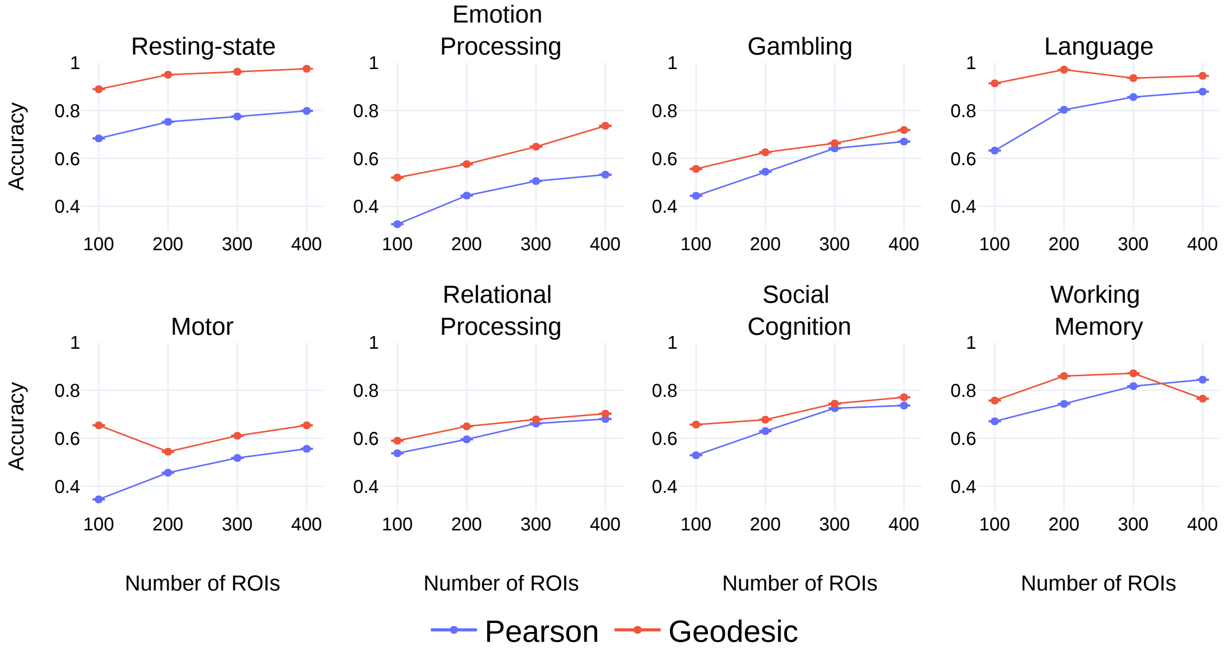

Fig. S5: Participant identification accuracy as a function of the number of ROIs. Here, training and testing data are from the same condition. Error bars indicate standard error of the mean across the bootstrap iterations.

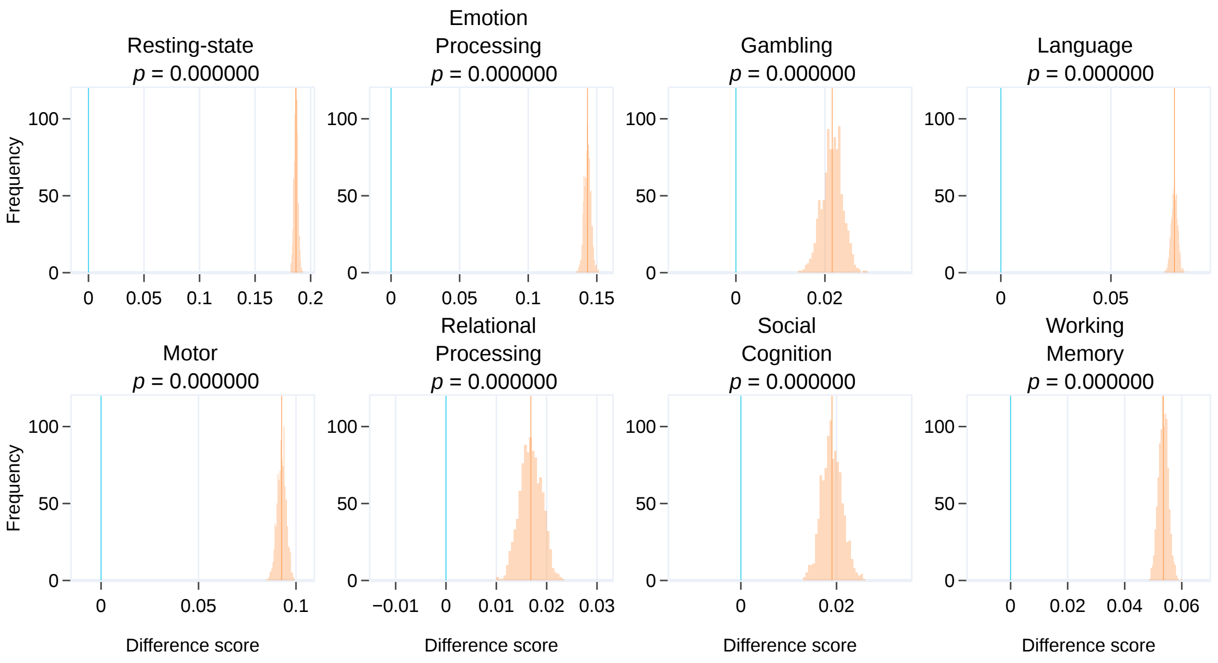

Fig. S6: *Whole-cortex FCs with full time course lengths:* Comparison of identification accuracy based on geodesic distance and Pearson dissimilarity for each condition. Identification was based on whole-cortex FCs. Here, full time course lengths were used (see Section 2.1). For each condition, the distributions shown in orange represent the difference between the mean participant identification accuracy using the geodesic distance and Pearson dissimilarity across the outer bootstrap iterations (see Section 2.7). The orange line indicates the mean of the difference distribution and the blue line indicates zero difference.

bioRxiv preprint first posted online Jul. 2, 2019; doi: http://dx.doi.org/10.1101/687830. The copyright holder for this preprint (which was not peer-reviewed) is the author/funder, who has granted bioRxiv a license to display the preprint in perpetuity. It is made available under a CC-BY-NC-ND 4.0 International license.

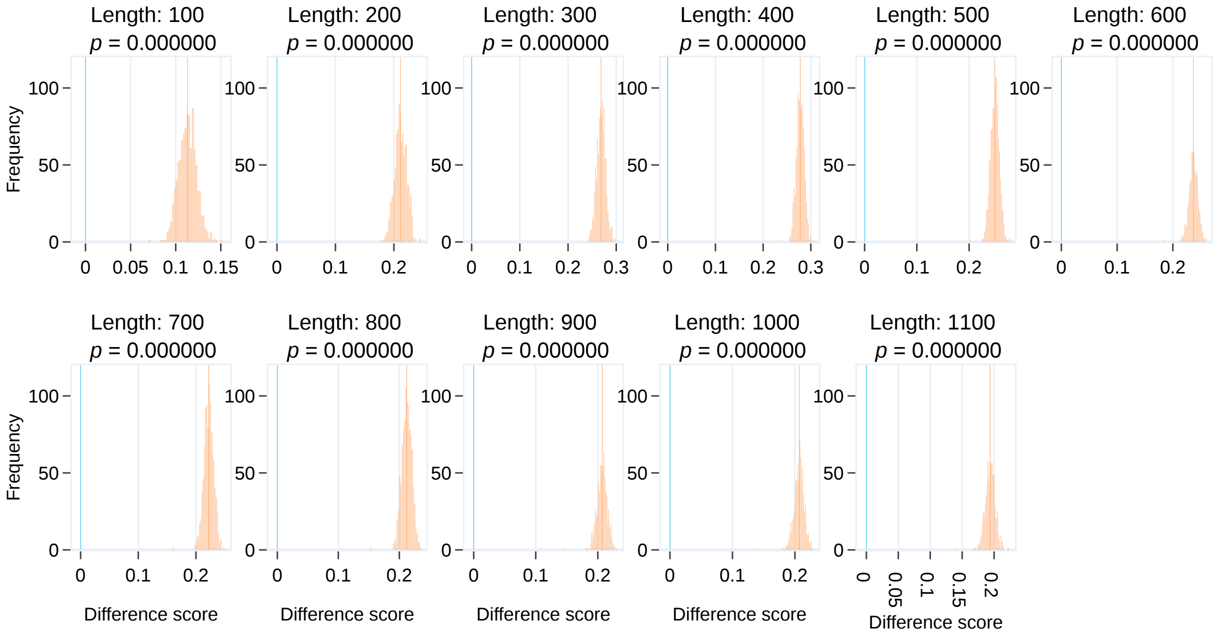

Fig. S7: *Identification accuracy and time course length:* Comparison of identification accuracy based on geodesic distance and Pearson dissimilarity for various time course lengths. Since *resting-state* data had the highest time course length, smaller segments of various lengths were extracted (see Section 2.7.1). Identification was based on whole-cortex FCs. For each segment length, the distributions shown in orange represent the difference between the mean participant identification accuracy using the geodesic distance and Pearson dissimilarity across the outer bootstrap iterations (see Section 2.7). The orange line indicates the mean of the difference distribution and the blue line indicates zero difference.

bioRxiv preprint first posted online Jul. 2, 2019; doi: http://dx.doi.org/10.1101/687830. The copyright holder for this preprint (which was not peer-reviewed) is the author/funder, who has granted bioRxiv a license to display the preprint in perpetuity. It is made available under a CC-BY-NC-ND 4.0 International license.

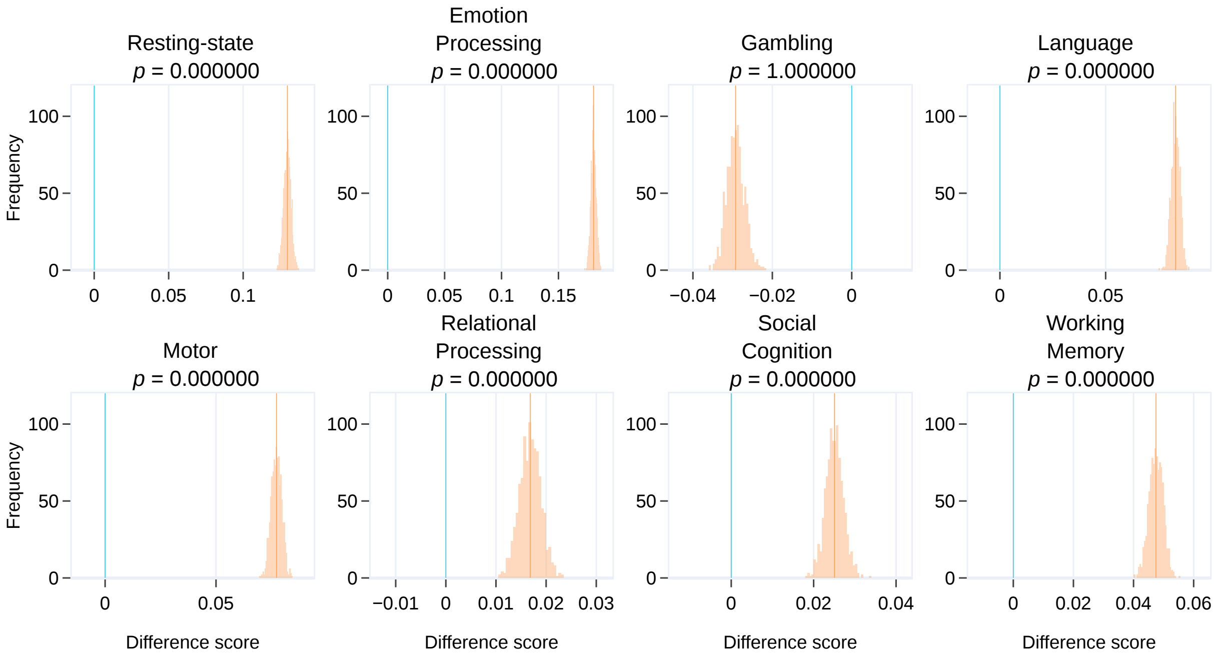

Fig. S8: *Whole-cortex FCs with trimmed time course lengths:* Comparison of identification accuracy based on geodesic distance and Pearson dissimilarity for each condition. Identification was based on whole-cortex FCs. Data for each condition were trimmed such that they all had the same time course length (of 138; see Section 3.5). For each condition, the distributions shown in orange represent the difference between the mean participant identification accuracy using the geodesic distance and Pearson dissimilarity across the outer bootstrap iterations (see Section 2.7). The orange line indicates the mean of the difference distribution and the blue line indicates zero difference.

bioRxiv preprint first posted online Jul. 2, 2019; doi: http://dx.doi.org/10.1101/687830. The copyright holder for this preprint (which was not peer-reviewed) is the author/funder, who has granted bioRxiv a license to display the preprint in perpetuity. It is made available under a CC-BY-NC-ND 4.0 International license.

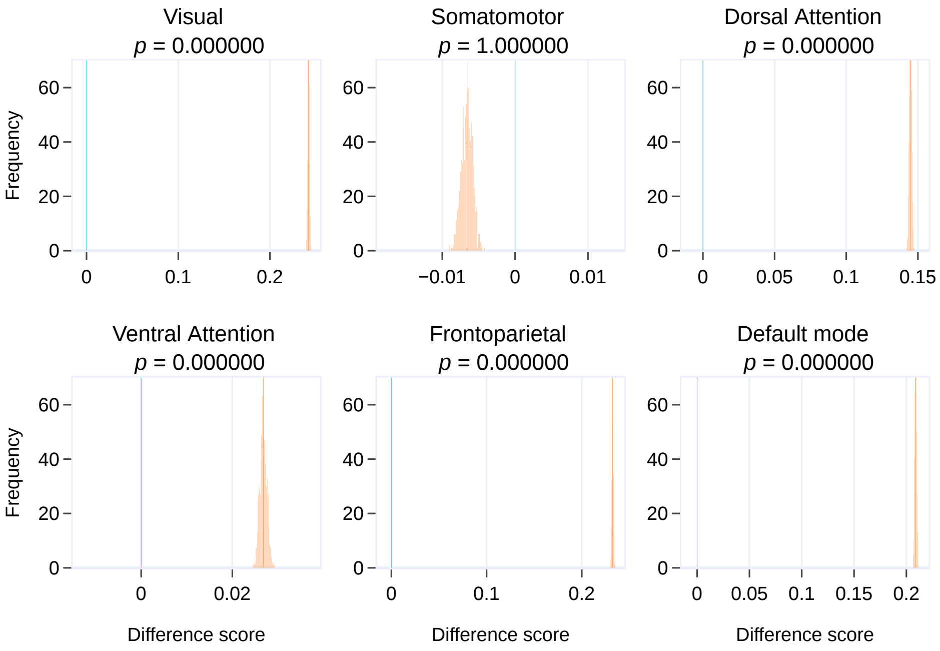

Fig. S9: *Subnetwork FCs with trimmed time course lengths:* Comparison of identification accuracy based on geodesic distance and Pearson dissimilarity for each subnetwork. Identification was based on subnetwork FCs. Data for each condition were trimmed such that they had the same time course length (of 138; see Section 3.5). For each subnetwork, difference scores were averaged across all conditions. The distributions shown in orange represent the difference between the mean participant identification accuracy using the geodesic distance and Pearson dissimilarity across the outer bootstrap iterations (see Section 2.7). The orange line indicates the mean of the difference distribution and the blue line indicates zero difference.

bioRxiv preprint first posted online Jul. 2, 2019; doi: http://dx.doi.org/10.1101/687830. The copyright holder for this preprint
(which was not peer-reviewed) is the author/funder, who has granted bioRxiv a license to display the preprint in perpetuity.
It is made available under a CC-BY-NC-ND 4.0 International license.

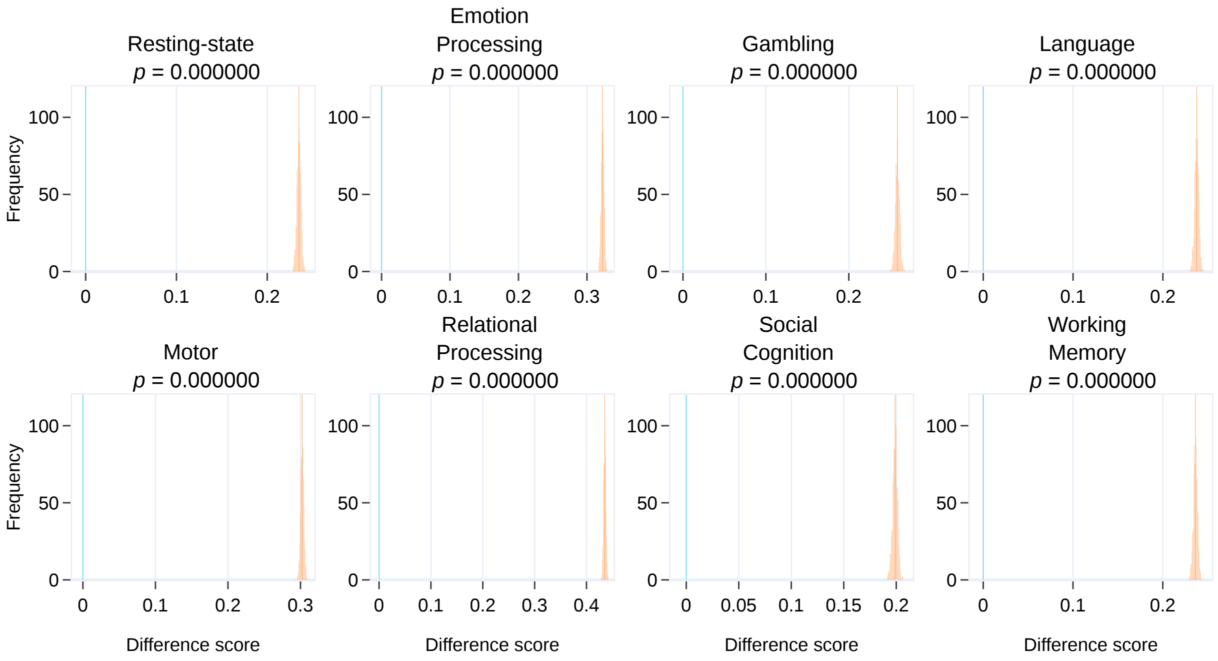

Fig. S10: *Subnetworks of the same size:* Comparison of identification accuracy using `dorsal attention` and `ventral attention` subnetwork FCs for each condition. The geodesic distance measure was used for identification. The two subnetworks were of identical size for the 300 ROIs parcellation (see Table 2. Data for each condition were trimmed such that they all had the same time course length (of 138; see Section 3.5). For each condition, the distributions shown in orange represent the difference between the mean participant identification accuracy based on the two subnetworks across the outer bootstrap iterations (see Section 2.7). The orange line indicates the mean of the difference distribution and the blue line indicates zero difference.

bioRxiv preprint first posted online Jul. 2, 2019; doi: http://dx.doi.org/10.1101/687830. The copyright holder for this preprint (which was not peer-reviewed) is the author/funder, who has granted bioRxiv a license to display the preprint in perpetuity. It is made available under a CC-BY-NC-ND 4.0 International license.

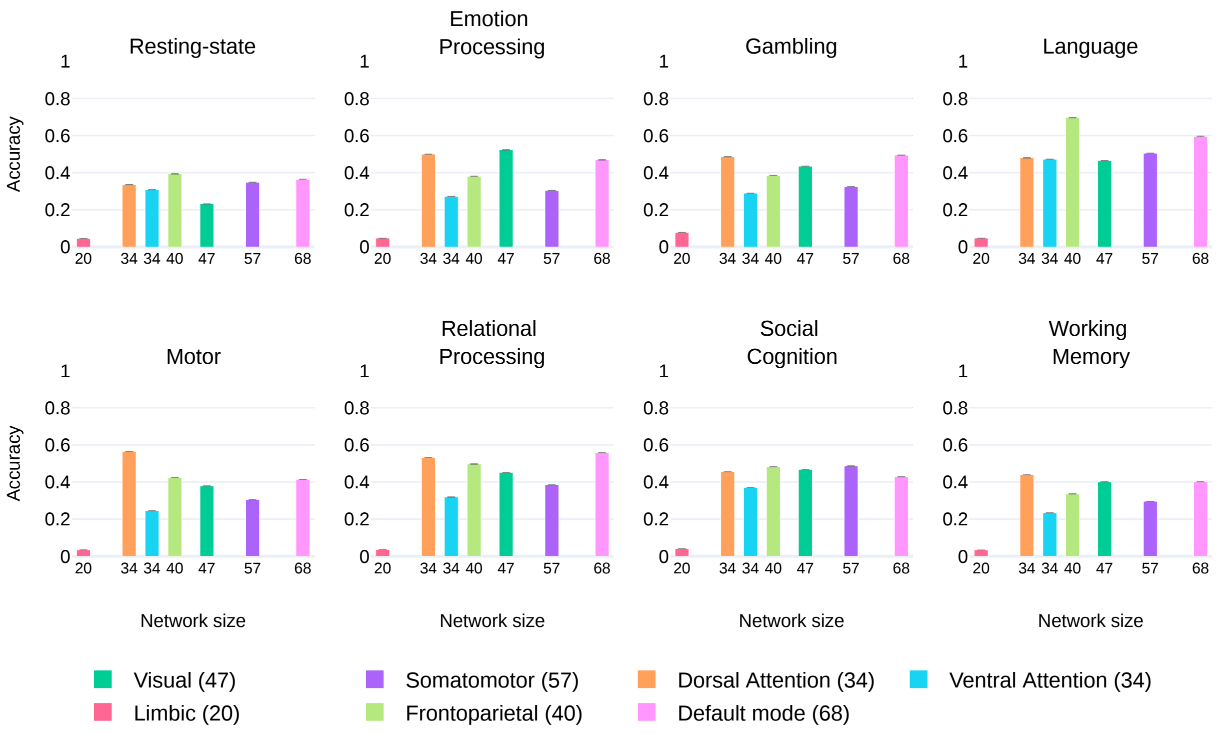

Fig. S11: Participant identification accuracy plotted against subnetwork size for each condition (Pearson dissimilarity). The size of the subnetwork (the number of ROIs) is also indicated in the inset. The error bars represent standard error of the mean across bootstrap iterations.

bioRxiv preprint first posted online Jul. 2, 2019; doi: http://dx.doi.org/10.1101/687830. The copyright holder for this preprint (which was not peer-reviewed) is the author/funder, who has granted bioRxiv a license to display the preprint in perpetuity. It is made available under a CC-BY-NC-ND 4.0 International license.

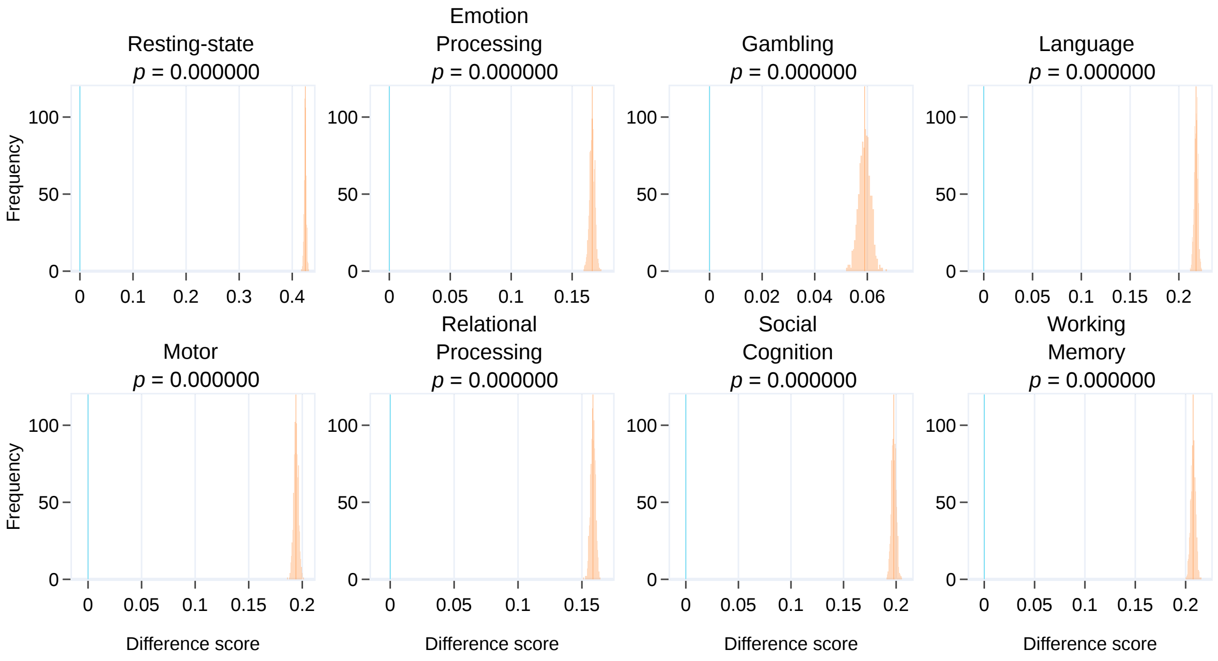

Fig. S12: *Combined subnetwork FCs with trimmed time course lengths:* Comparison of identification accuracy based on geodesic distance and Pearson dissimilarity for each condition. Identification was based on combined subnetwork FCs (see Section 3.7). Data for each condition were trimmed such that they all had the same time course length (of 138; see Section 3.5). For each condition, the distributions shown in orange represent the difference between the mean participant identification accuracy using the geodesic distance and Pearson dissimilarity across the outer bootstrap iterations (see Section 2.7). The orange line indicates the mean of the difference distribution and the blue line indicates zero difference.

bioRxiv preprint first posted online Jul. 2, 2019; doi: http://dx.doi.org/10.1101/687830. The copyright holder for this preprint (which was not peer-reviewed) is the author/funder, who has granted bioRxiv a license to display the preprint in perpetuity. It is made available under a CC-BY-NC-ND 4.0 International license.

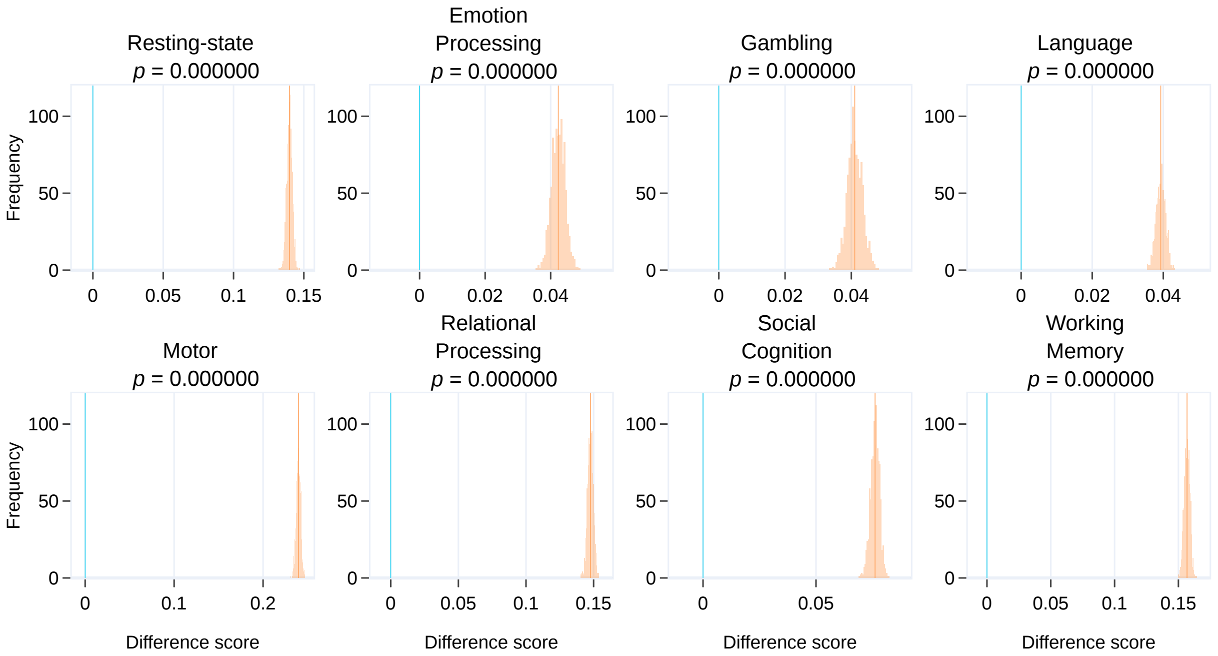

Fig. S13: *Combined subnetwork vs frontoparietal:* Comparison of identification accuracy based on combined subnetwork FCs (see Section 3.7) and `frontoparietal` subnetwork FCs (part of the combined subnetwork) for each condition. The geodesic distance measure was used for identification. Data for each condition were trimmed such that they all had the same time course length (of 138; see Section 3.5). For each condition, the distributions shown in orange represent the difference between the mean participant identification accuracy based on combined subnetwork FCs and `frontoparietal` FCs across the outer bootstrap iterations (see Section 2.7). The orange line indicates the mean of the difference distribution and the blue line indicates zero difference.

bioRxiv preprint first posted online Jul. 2, 2019; doi: http://dx.doi.org/10.1101/687830. The copyright holder for this preprint (which was not peer-reviewed) is the author/funder, who has granted bioRxiv a license to display the preprint in perpetuity. It is made available under a CC-BY-NC-ND 4.0 International license.

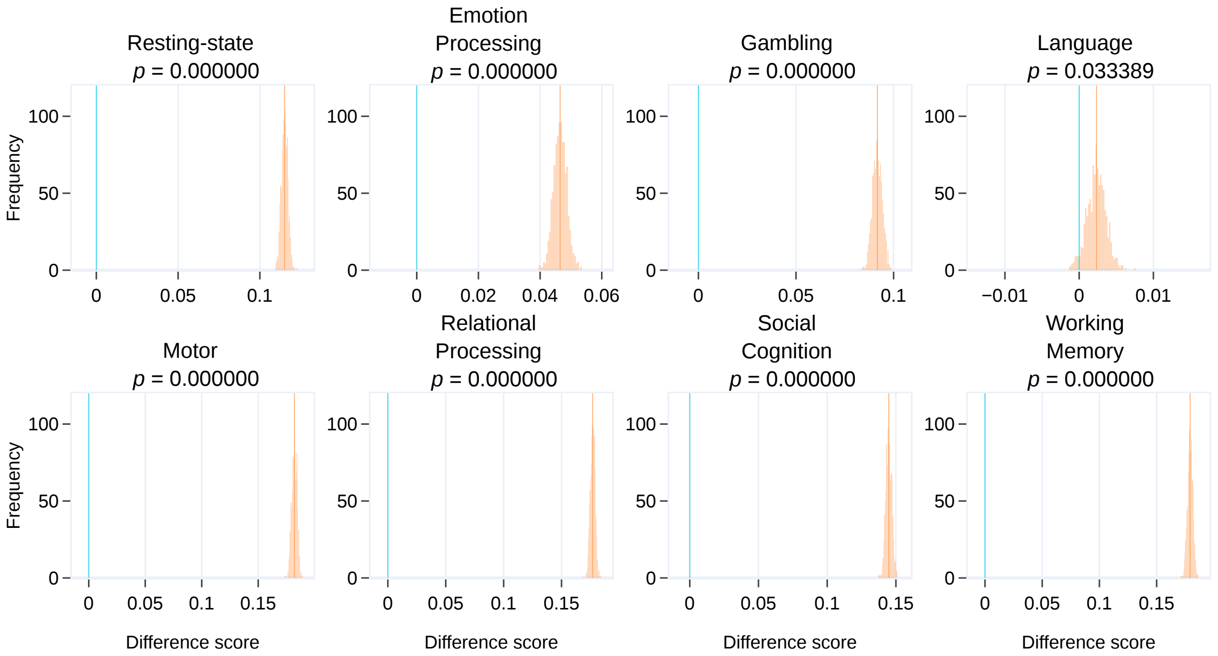

Fig. S14: *Combined subnetwork vs default mode:* Comparison of identification accuracy based on combined subnetwork FCs (see Section 3.7) and `default mode` subnetwork FCs (part of the combined subnetwork) for each condition. The geodesic distance measure was used for identification. Data for each condition were trimmed such that they all had the same time course length (of 138; see Section 3.5). For each condition, the distributions shown in orange represent the difference between the mean participant identification accuracy based on combined subnetwork FCs and `default mode` FCs across the outer bootstrap iterations (see Section 2.7). The orange line indicates the mean of the difference distribution and the blue line indicates zero difference.

bioRxiv preprint first posted online Jul. 2, 2019; doi: http://dx.doi.org/10.1101/687830. The copyright holder for this preprint (which was not peer-reviewed) is the author/funder, who has granted bioRxiv a license to display the preprint in perpetuity. It is made available under a CC-BY-NC-ND 4.0 International license.

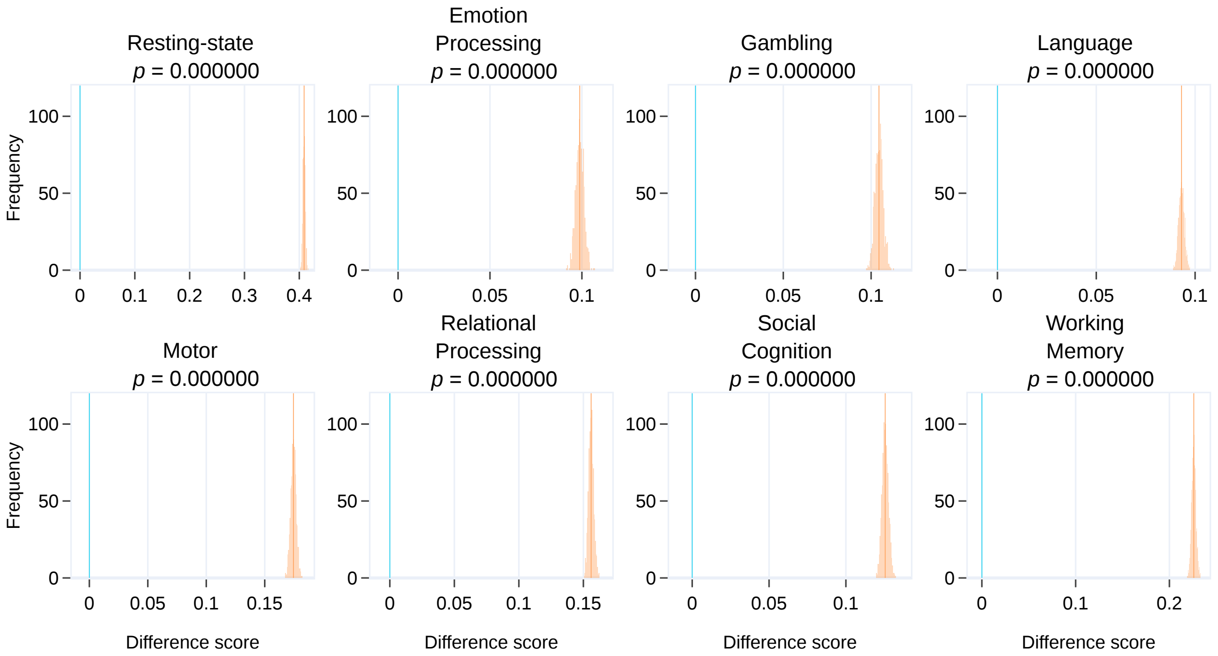

Fig. S15: *Combined subnetwork vs whole-cortex FCs:* Comparison of identification accuracy based on combined subnetwork FCs (see Section 3.7) and whole-cortex FCs for each condition. The geodesic distance measure was used for identification. Data for each condition were trimmed such that they all had the same time course length (of 138; see Section 3.5). For each condition, the distributions shown in orange represent the difference between the mean participant identification accuracy based on combined subnetwork FCs and whole-cortex FCs across the outer bootstrap iterations (see Section 2.7). The orange line indicates the mean of the difference distribution and the blue line indicates zero difference.
